# A high-throughput single-molecule platform to study DNA supercoiling effect on protein–DNA interactions

**DOI:** 10.1093/nar/gkaf581

**Published:** 2025-06-28

**Authors:** Huijin Lee, Fahad Rashid, Jihee Hwang, James A London, Richard Fishel, James M Berger, Sua Myong, Taekjip Ha

**Affiliations:** Howard Hughes Medical Institute and Programs in Cellular and Molecular Medicine, Boston Children’s Hospital, Boston, MA 02115, United States; Department of Biophysics and Biophysical Chemistry, Johns Hopkins School of Medicine, Baltimore, MD 21205, United States; Department of Biophysics and Biophysical Chemistry, Johns Hopkins School of Medicine, Baltimore, MD 21205, United States; Programs in Cellular and Molecular Medicine, B oston Children’s Hospital, Boston, MA 02115, United States; Department of Pediatrics, Harvard Medical School, Boston, MA 02115, United States; Department of Cancer Biology and Genetics, The Ohio State University Wexner Medical Center, Columbus, OH 43210, United States; Department of Cancer Biology and Genetics, The Ohio State University Wexner Medical Center, Columbus, OH 43210, United States; Department of Biophysics and Biophysical Chemistry, Johns Hopkins School of Medicine, Baltimore, MD 21205, United States; Programs in Cellular and Molecular Medicine, B oston Children’s Hospital, Boston, MA 02115, United States; Department of Pediatrics, Harvard Medical School, Boston, MA 02115, United States; Howard Hughes Medical Institute and Programs in Cellular and Molecular Medicine, Boston Children’s Hospital, Boston, MA 02115, United States; Department of Biophysics and Biophysical Chemistry, Johns Hopkins School of Medicine, Baltimore, MD 21205, United States; Department of Pediatrics, Harvard Medical School, Boston, MA 02115, United States

## Abstract

DNA supercoiling significantly influences DNA metabolic pathways. To examine its impact on DNA–protein interactions at the single-molecule level, we developed a highly efficient and reliable protocol to modify plasmid DNA at specific sites, allowing us to label plasmids with fluorophores and biotin. We then induced physiological levels of negative or positive supercoiling in these plasmids using gyrase or reverse gyrase, respectively. By comparing supercoiled DNA with relaxed circular DNA, we assessed the effects of supercoiling on CRISPR–Cas9 and the mismatch repair protein MutS. We found that negative DNA supercoiling exacerbates off-target effects in DNA unwinding by Cas9. For MutS, we observed that both negative and positive DNA supercoiling enhance the binding interaction between MutS and a mismatched base pair but do not affect the rate of ATP-induced sliding clamp formation. These findings not only underscore the versatility of our protocol but also open new avenues for exploring the intricate dynamics of protein–DNA interactions under the influence of supercoiling.

## Graphical Abstract

**Graphical Abstract:**
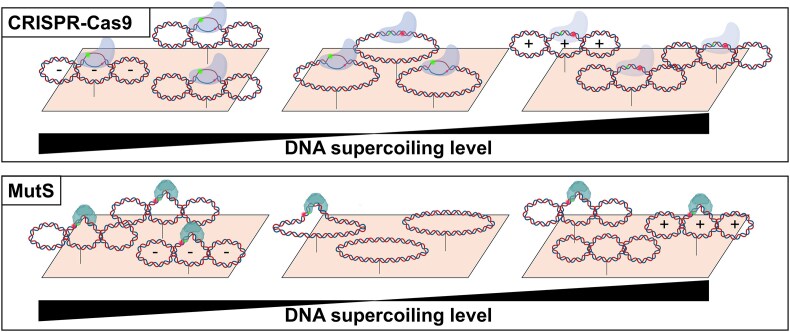

## Introduction

DNA supercoiling, the twisting and winding of the double helix structure beyond its relaxed state, is a fundamental mechanism for regulating numerous biological processes [1, 2]. From the compact packaging of genetic material within the confines of the cell nucleus to the dynamic modulation of gene activity [3], DNA supercoiling functions as a versatile molecular switch, controlling vital cellular functions [4]. It regulates DNA accessibility [5], replication, and transcription, profoundly impacting gene expression and genome stability. For example, RNA polymerase generates positive supercoiling ahead of the transcription machinery and negative supercoiling behind it [6–8]. These accumulated supercoiling states must be resolved by topoisomerases, and failure to do so can lead to transcriptional arrest [9]. Similarly, the replisome generates positive supercoiling in front of it [10–12] and topoisomerases are required for proper elongation by the replisome and chromosome segregation during cell division [13, 14].

To study how DNA supercoiling affects protein behavior, various techniques have been employed. Traditional methods like electrophoresis differentiate DNA molecules based on their migration speed, which varies according to their supercoiling status [15, 16]. However, electrophoresis does not allow real-time observation of functionally relevant dynamics. Single-molecule techniques provide additional insights by allowing precise control of DNA supercoiling while enabling the observation of protein–DNA dynamics in real time. In magnetic tweezers, a single linear double-stranded DNA molecule is tethered at both ends, with one extremity linked to a paramagnetic bead and the other anchored to the glass surface of a flow cell [17, 18]. Tethering to each surface is achieved through multiple bonds, preventing the DNA from swiveling around the attachment points. By rotating the bead via manipulating a magnet above the sample chamber, magnetic tweezers can control torque and twist on DNA in real time [19]. Magnetic tweezers allow for precise application of force and torque, making it possible to study the dynamic behavior of DNA–protein interactions under varying supercoiling conditions [5, 20, 21]. Additionally, by attaching a fluorescent rotor bead to the side of DNA, it is possible to measure the torque and twist exerted on DNA [22]. As the DNA swivels, the rotor bead also swivels, and its associated rotation is observed through fluorescence imaging. This method allows for real-time observation of the rotational dynamics of DNA [23]. Optical tweezers offer higher temporal resolution compared to magnetic tweezers, making them suitable for studying fast dynamics [24]. Typically, optical tweezers do not have the ability to control DNA supercoiling with two notable exceptions: using a birefringent particle instead of a bead [25], or employing DNA overstretching followed by strand/bead untethering and retethering [26]. However, optical tweezers can only manipulate one molecule at a time when introducing supercoiling into DNA, resulting in inherently low throughput. Another approach, convex lens-induced confinement (CLiC) microscopy, enables the study of DNA dynamics without surface tethering and offers a more native-like environment, though it has not yet been widely applied for high-throughput protein–DNA interaction studies [27–30].

Here, we introduce a single-molecule approach to study the effects of DNA supercoiling on protein–DNA interactions with high throughput. By implementing single-molecule total internal reflection fluorescence (smTIRF) microscopy and utilizing an enzyme-based site-specific DNA modification method, originally reported by the Seidel laboraotry [31], and subsequently extended to circular, supercoiled DNA by the Leng laboratory [80, 81]. We established a single-molecule fluorescence resonance energy transfer (FRET)-based high-throughput assay [32]. This platform allowed us to investigate the effects of DNA supercoiling on two different systems: CRISPR–Cas9 and DNA mismatch repair (MMR). In the CRISPR–Cas9 system, we monitored real-time DNA unwinding kinetics as a function of supercoiling in the presence of mismatched sequences. Our results suggest that negative DNA supercoiling enhances DNA unwinding by the nuclease-deficient Cas9 (dCas9) in complex with guide RNA (gRNA) (dCas9–gRNA), in the presence of mismatches within either the protospacer adjacent motif-proximal or -distal regions. Furthermore, we observed that the dynamics of MutS, a protein that recognizes a mismatch to initiate MMR, depend on DNA supercoiling state. We found that the initial binding between MutS and a mismatched base pair can be enhanced by DNA supercoiling in both directions.

## Materials and methods

### Cloning

Plasmids pUC19HL_MutS and pUC19HL_CRI were prepared by introducing two BbvCI nick sites and one BsmI restriction site to plasmid pUC19. A BsmI site was introduced into the plasmid by site-directed mutagenesis using the Q5 Site-Directed Mutagenesis Kit (NEB, E0554), following the manufacturer’s protocol. The primers used were 5′-ATGCCAAAATCCCTTAACGTG-3′ and 5′-TCATGAGATTATCAAAAAGGATC-3′. Vector DNA was prepared by digestion with EcoRI (NEB, R3101S, 2 units/μg DNA) and HindIII (NEB, R3104S, 2 units/μg DNA) of plasmid pUC19 (NEB, N3041S) supplemented with rCutSmart Buffer (NEB, B6004S) for 15 min at 37°C. The DNA was purified using PCR (polymerase chain reaction) spin column (Qiagen, 28104). Insert DNA for pUC19HL_MutS was prepared by annealing two 5′-phosphorylated oligonucleotides 5′-AGCTTCCTCAGCTTAATACGACTCACTATAGGCCAATACAAGAGCTTCATCCTCAGCG-3′ and 5′-AATTCGCTGAGGATGAAGCTCTTGTATTGGCCTATAGTGAGTCGTATTAAGCTGAGGA-3′. Insert DNA for pUC19HL_CRI was prepared by annealing two 5′-phosphorylated oligonucleotides 5′-AGCTTCCTCAGCCCAGCGTCTCATCTTTATACATCAGCAGAGATTTCTGCTGTGCAACCTCAGCG-3′ and 5′-AATTCGCTGAGGTTGCACAGCAGAAATCTCTGCTGATGTATAAAGATGAGACGCTGGGCTGAGGA-3′. The vector DNA and insert DNA were then incubated with T4 DNA ligase at room temperature for 10 min, followed by inactivation at 60°C for 10 min. The mixture was added to competent DH5α *Escherichia coli* cells (NEB, C2988J) and incubated at 42°C for 30 s for transformation. Transformed cells were cultured in 1 ml of LB broth and plated on LB agar plates supplemented with ampicillin. Several colonies were picked and grown in LB broth. Plasmids from each colony were extracted using a mini prep kit (Omega, D6942). The sequence was confirmed by Sanger sequencing (GENEWIZ from Azenta Life Sciences).

### Labeled oligonucleotides’ preparation

All DNA oligonucleotides were purchased from Integrated DNA Technologies (IDT). A thymine modified with an amine group through a C6 linker (amino-dT) was used for labeling. All oligonucleotide sequences and the location of amino modification and biotin are shown in Supplementary Table S1. The nucleotide (5 nmol) and sulfo-Cy3 NHS ester (Lumiprobe, 13320) or sulfo-Cy5 NHS ester (Lumiprobe, 11320) (250 nmol) were mixed in 0.1 M NaHCO_3_ solution. The mixture was incubated at 40°C overnight. The labeled nucleotides were precipitated by incubating with three times the volume of ethanol at −80°C for 1 h. The mixture was centrifuged at 20 000 × *g* at 4°C for 40 min. The remaining dyes were removed by carefully removing the supernatant and washing the pellet with 70% ethanol solution. The residual ethanol was dried in the air and the DNA pellet was suspended in nuclease-free water.

### Strand replacement

To create circular DNA with necessary modifications for single molecule fluorescence measurements of surface-immobilized molecules, we closely followed an enzyme-based site-specific DNA modification protocol originally developed by the Seidel laboratory [31], and subsequently extended to circular, supercoiled DNA by the Leng laboratory [80, 81]. Plasmid DNA was purified using QIAGEN Plasmid Plus Maxi Kit (QIAGEN, 12963). Plasmid DNA (5 μg) and labeled single-stranded oligo (50-fold excess relative to the DNA molarity) were mixed with either Nt.BbvCI (NEB, R0632) or Nb.BbvCI (NEB, R0631) (1.75 μl, 17.5 units) and rCutSmart buffer (supplied with enzyme) (5 μl) in a total volume of 50 μl. The mixture was incubated at 37°C for 3 h and then at 95°C for 5 min. The temperature decreased to 25°C slowly (0.2°C/min). The extra single-stranded oligo was removed using 50K Amicon (Millipore Sigma, UFC5050BK). The purified plasmid DNA (5 μg) was incubated with T4 DNA ligase (NEB, M0202) (2.5 μl, 1000 units) and T4 ligase reaction buffer (supplied with enzyme) (7.5 μl) in a total volume of 75 μl overnight at room temperature. After the incubation, DNA was purified using PCR spin columns (Qiagen, 28104). The second strand replacement was performed in the same manner as the first strand replacement, but the Amicon purification step was omitted. After annealing, the mixture was incubated with T4 DNA ligase (NEB, M0202) (2.5 μl, 1000 units), supplemented with dithiothreitol (DTT) (1 mM) and ATP (1 mM) in a total volume of 75 μl overnight at room temperature. The mixture was treated with T5 exonuclease (NEB, M0663) (5 μl, 50 units) for 30 min at 37°C. After the reaction, DNA was purified using PCR spin columns (Qiagen, 28104). DNA concentration and purity (*A*_260_/*A*_230_ and *A*_260_/*A*_280_) were confirmed by NanoDrop. The plasmids at each step were confirmed by 1% agarose gel.

### Gyrase preparation

Full-length GyrA and GyrB were cloned into pRSF vector with double His-tag and SUMO-tag at the N-terminus. The GyrA and GyrB constructs were transformed into Rosetta™(DE3)pLysS cells, grown in 2xYT media at 37°C, and expressed at OD 0.8 for 4 h with 0.5 mM isopropyl β-D-1-thiogalactopyranoside (IPTG). Cells were harvested and resuspended in Buffer A [50 mM HEPES, pH 8.0, 40 mM imidazole, pH 8.0, and 10% glycerol along with protease inhibitors (1 mM PMSF, 1 μg/ml pepstatin A, and 1 μg/ml leupeptin)] containing 1 M NaCl (A1000). To purify full-length GyrA and GyrB constructs, cells were thawed and lysed by adding lysozyme (2 mg/ml), incubating on ice for 30 min, and then passing through a HisTrap-HP 5-ml column. After washing with 10 column volumes (CV) of Buffer A1000, and further with 10 CV Buffer A containing 150 mM NaCl (A150), protein was eluted directly onto an A150-equilibrated HiTrap-Q 5-ml column. The column was washed with 10 CV of Buffer A150, and protein was eluted using a gradient of A150 and A1000. Peak fractions containing the protein were collected, and the His–SUMO tag was removed via overnight digestion at 4°C using SUMO protease. The mixture was then dialyzed against Buffer A1000 to remove excess imidazole. After tag removal, the cleaved tag and protease were separated from the protein by repassing the solution over a HisTrap column in Buffer A1000, and the flowthrough was concentrated. The protein was further purified by loading onto a HiLoad Superdex 200 column equilibrated with a buffer containing 500 mM KCl, 50 mM HEPES (pH 8.0), and 10% glycerol (S500). Peak fractions, assessed using sodium dodecyl sulfate–polyacrylamide gel electrophoresis (SDS–PAGE), were collected, concentrated, and adjusted to 20% glycerol to act as a cryoprotectant prior to freezing in liquid nitrogen.

### Plasmid DNA preparation with different DNA supercoiling status

Negatively supercoiled DNA was prepared by incubating with gyrase (five-fold excess relative to the DNA molarity) in 30 mM Tris–HCl (pH 7.8), 200 mM potassium glutamate, 1 mM tris(2-chloroethyl) phosphate (TCEP), 10 mM Mg(OAc)_2_, 10% glycerol, and 5 mM ATP at 37°C for 1 h. The gyrase was inactivated at 65°C for 20 min. Positively supercoiled DNA was prepared by incubating with 9°N reverse gyrase (5 units/μg DNA, M0200, provided by New England Biolabs) in 35 mM Tris–HCl (pH 7.5 at 25°C), 24 mM KCl, 4 mM MgCl_2_, 2 mM DTT, 1.75 mM spermidine, 0.1 mg/ml bovine serum albumin (BSA), and 6.5% glycerol at 80°C for 30 min. In both reactions, T5 exonuclease (5 units/μg DNA, NEB, M0363) was added to the mixture and incubated at 37°C for 30 min to digest the remaining nicked DNA. Nicked DNA was prepared by BsmI (NEB, R0134S) (2 units/μg DNA) at 65°C for 1 h. All plasmid DNAs were purified using PCR spin column (Qiagen, 28104).

### Determination of the DNA superhelical density by 2D gel electrophoresis

To determine the superhelical density of supercoiled DNA samples, topoisomers were separated using 2D electrophoresis on a 2% agarose 1× TAE gel. For negatively supercoiled DNA, the first dimension contained 3 μg/ml chloroquine, while no chloroquine was added for positively supercoiled DNA. The gel was run at 2.2 V/cm for 20 h. The gel was then equilibrated in 1× TAE buffer containing 20 μg/ml chloroquine for negatively supercoiled DNA and 20 μg/ml chloroquine for positively supercoiled DNA. It was then rotated 90^0^ and run for an additional 20 h at 2.2 V/cm. Chloroquine was subsequently removed from the gels by soaking in TAE buffer, and topoisomers were visualized using SYBR Gold staining.

The extent of supercoiling was approximated using the formula:


\begin{eqnarray*}
\sigma = \Delta {\mathrm{Lk}}/{\mathrm{L}}{{\mathrm{k}}_0},
\end{eqnarray*}

where ΔLk = Lk − Lk_0_ is the change in linking number, Lk is the actual linking number of the DNA, and Lk_0_ is the linking number of relaxed DNA. The Lk_0_ value was calculated based on a DNA length of 2800 bp.

### Microscopy and data acquisition for single-molecule assays

Microscopy was conducted using a Nikon Eclipse Ti microscope equipped with a custom prism-type TIRFM module, controlled by home-built software (smCamera 2.0) [33]. A Nikon 60×/1.27 NA objective (CFI Plan Apo IR 60XC WI) was utilized. Illumination was provided by solid-state lasers (Coherent, 641 nm), which were combined and coupled to an optical fiber. Emission signals were collected through long-pass filters (T540LPXR UF3, T635LPXR UF3, T760LPXR UF3) and a custom laser-blocking notch filter (ZET488/543/638/750M) from Chroma. Images were captured with an electron-multiplying charge-coupled device (Andor iXon 897).

### CRISPR–Cas9

#### *dCas9*–*g*RNA complex preparation

For gRNA assemble, CRISPR RNA (crRNA) (IDT) and trans-activating CRISPR RNA (tracrRNA) (IDT, 1072532) were diluted in nuclease-free IDTE buffer (supplemented with tracrRNA) and mixed at 1:1 ratio in nuclease-free water (final concentration 10 μM). The mixture was heated at 95°C for 5 min and cooled to room temperature (1°C/min). The dCas9–gRNA complex was assembled by mixing guide RNA and dCas9 protein (IDT, 1081066) at a ratio of 2:1 in Cas9 dilution buffer (30 mM 2-(4-(2-hydroxyethyl)-1-piperazinyl)-ethanesulfonic acid (HEPES), 150 mM KCl, pH 7.5). RNA sequences are available in Supplementary Table S2.

#### Single-molecule fluorescence imaging and quantification

Detailed methods of smFRET data acquisition and analysis were described previously [34]. For the DNA unwinding assay, 200 pM of Cy3-, Cy5-, and biotin-labeled plasmid DNA was immobilized on the polyethylene glycol-passivated flow chamber surface (Nano Surface Sciences) using NeutrAvidin–biotin interaction. All the imaging were carried out at room temperature in Cas9–gRNA reaction buffer supplemented with oxygen scavenging reagents and Trolox [20 mM Tris–HCl, pH 8, 100 mM KCl, 5 mM MgCl_2_, 5% (v/v) glycerol, 0.2 mg/ml BSA, 1 mg/ml glucose oxidase, 0.03 mg/ml catalase, saturated Trolox (> 5 mM), and 0.8% (w/v) dextrose]. All movies were recorded with a 50 ms time resolution. Before the addition of the dCas9–gRNA complex, short movies of the first 10 frames under Cy3 excitation and the last 10 frames under Cy5 excitation were recorded at 10 different imaging views. Following the addition of 10 nM dCas9–gRNA complex with a mismatch on PAM-proximal region, the same format of short movies was captured at each of the following time points: 1, 2, 3, 4, 7, 10, 13, 20, 30, 45, and 60 min. All data points were used for fitting, but the plot was trimmed to exclude the data points of 30, 45, and 60 min. Recording 10 movies required ∼20 s and yielded data from around 5000 molecules. In the case of dCas9–gRNA with three consecutive mismatches on PAM-distal region, the movies composed of initial and last 10 frames under Cy5 excitation and 980 frames under Cy3 excitation were recorded from five different fields of view.

#### DNA unwinding kinetics analysis

The first 10 frames were used to calculate FRET efficiency and the last 10 frames were used to filter the fluorescent signal from inactive or missing acceptor. Five frames from third to seventh frames of each molecule’s FRET trajectories were selected as data points to construct FRET histograms. The FRET histograms were fitted using a sum of three Gaussian functions. The intersection points of the Gaussian curves were determined to divide the population into two groups by FRET. For dCas9–gRNA with a single mismatch on PAM-proximal region, molecules exhibiting FRET values between 0.14 and 0.47 were classified as DNA unwound by the dCas9–gRNA complex. Those with FRET values exceeding 0.14 were categorized as total DNA molecules. In the case of dCas9–gRNA with three consecutive mismatches in PAM-distal region, those with FRET values exceeding 0.18 were categorized as total DNA molecules and those with FRET between 0.18 and 0.51 were classified as DNA unwound. For the dwell time analysis, the FRET traces with dynamics were collected. The FRET dynamics as a function of time was characterized into two states using a hidden Markov model and the dwell times for each state were collected by ebFRET [35].

### MutS

#### Preparation of fluorescently labeled MutS

*Escherichia coli* MutS was cloned with an N-terminal hexa-histidine and sortase (LPETG) tag into a pET expression plasmid. MutS was expressed in BL21-AI cells with 1 l of culture. The culture was grown at 37°C until it reached an OD_600_ of 0.3. The temperature was lowered to 16°C and 0.2% l-(+)-arabinose was added. After 30 min, 0.05 mM IPTG was added and the culture grew for 16 h. The cells were collected and resuspended in Buffer A (25 mM HEPES, pH 7.8, 10% glycerol, 0.8 μg/ml pepstatin, 1 μg/ml leupeptin, and 87 μg/ml phenylmethylsulfonyl fluoride (PMSF)) containing 300 mM NaCl and 20 mM imidazole. The resuspended cell pellet was flash frozen and stored at −80°C. For the purification of MutS, all steps were carried out at 4°C. The cell pellet was thawed and lysed by sonication. The lysate was clarified by centrifuging at 120 000 × *g* for 1 h at 4°C. The supernatant was then applied to a 3-ml Ni-NTA (Qiagen) column. The Ni-NTA column was washed with 10 CV of Buffer A containing 800 mM NaCl and 20 mM imidazole, followed by 5 CV of Buffer A containing 90 mM NaCl and 20 mM imidazole. The Ni-NTA column was then eluted directly onto a tandem 3-ml Heparin-Sepharose (Cytiva) column with 5 CV of Buffer A containing 90 mM NaCl and 200 mM imidazole, washed with 10 CV of Buffer B (25 mM HEPES, pH 7.8, and 10% glycerol) containing 100 mM NaCl, and eluted with a 20 ml linear gradient of Buffer B containing 100 mM to 1 M NaCl. Peak fractions were analyzed by PAGE and combined. The combined MutS fractions were fluorescently labeled using a sortase transpeptidase. A total of 13 nmol of MutS was combined with 52 nmol of purified *Staphylococcus aureus* sortase 5 M and 130 nmol of purified Cy3-CLPETGG-labeled peptide. The labeling reaction was carried out at 4°C for 1 h in Buffer B containing 10 mM CaCl_2_ and 300 mM NaCl. The reaction was then stopped by the addition of 20 mM ethylene glycol-bis(β-aminoethyl ether)-N,N,N',N'-tetraacetic acid (EGTA). The excess label was removed by passing the reaction mix through a 10K MWCO Zeba spin desalting column (Thermo Scientific), followed by application onto a 1-ml Heparin-Sepharose column. Bound material was step eluted with Buffer C (25 mM HEPES, pH 7.8, 600 mM NaCl, 10% glycerol, 0.1 mM ethylenediaminetetraacetic acid (EDTA), 1 mM DTT, 0.8 μg/ml pepstatin, 1 μg/ml leupeptin, and 87 μg/ml PMSF), which removed the sortase protein and concentrated the MutS. Peak fractions were dialyzed into storage buffer (25 mM HEPES, pH 7.8, 150 mM NaCl, 20% glycerol, 0.1 mM EDTA, and 1 mM DTT), flash frozen, and stored at −80°C. We determined a 48% MutS monomer labeling efficiency.

#### Single-molecule fluorescence imaging and quantification

For the MutS binding assay, Cy5-labeled plasmid DNA containing single GT mismatch was immobilized on a polyethylene glycol-passivated flow chamber surface via NeutrAvidin–biotin interaction. Cy3-labeled *E. coli* MutS (2 nM) was incubated with 1 mM of ADP or ATP in MutS reaction buffer [20 mM Tris–HCl, pH 7.5, 5 mM MgCl_2_, 100 mM potassium glutamate, 0.1 mM DTT, 0.2 mg/ml acetylated BSA (Invitrogen, AM2614), and 0.0025% Tween 20 (Bio-Rad, 170-6531)], supplemented with oxygen-scavenging system [2.5 mM 3,4-dihydroxybenzoic acid (PCA; Sigma, 37580-25G-F) and 50 nM recombinant protocatechuate 3,4-dioxygenase from bacteria (rPCD or PCD) (OYC, 46852004)], and saturated Trolox (>5 mM). All movies were recorded at room temperature. In the presence of ADP, long movies consisting of the first 10 frames under Cy5 excitation, followed by 980 frames under Cy3 excitation and 10 frames under Cy5 excitation were recorded at three different imaging views with a time resolution of 200 ms. Under ATP condition, long movies consisting of the first 10 frames under Cy5 excitation, followed by 1480 frames under Cy3 excitation and 10 frames under Cy5 excitation were recorded at three different imaging views with a time resolution of 50 ms. The first 10 frames were used to select DNA.

#### Enzyme kinetics analysis

The dwell times were collected manually using custom MATLAB scripts. In ATP condition, the dwell times of high FRET (*t*_SB_) both followed by low FRET and not followed were collected manually using custom MATLAB scripts. The dwell time was plotted in 1 − CDF (cumulative distribution function) plot and fitted to single exponential decay function (equation 1, where *t* is the dwell time, *y*_0_ is baseline offset, and *A*_1_ is initial value of 1 − CDF at *t*= 0) to obtain the rate constant (*k*).


(1) \begin{eqnarray*}
{{y\;}} = {{\;}}{y_0} + {{\;}}{A_1}{{\rm e}^{ - kt}}.
\end{eqnarray*}

This rate constant is sum of two rate constants, *k*_−1_ (dissociation from mismatch region) and *k*_2_ (conformational change from initial binding to sliding clamp) (equation 2).


(2) \begin{eqnarray*}
k = {k_{ - 1}} + {k_2}.
\end{eqnarray*}

The branch ratio is defined as the number of sliding clamp formations (*N*_2_) divided by the number of specific binding events not followed by sliding clamp (*N*_−1_). By setting the equation (3), the *k*_−1_ and *k*_2_ were determined.


(3) \begin{eqnarray*}
\frac{{{N_2}}}{{{N_{ - 1}}}} = \frac{{{k_2}}}{{{k_{ - 1}}}}.
\end{eqnarray*}

All experiments were performed more than three times, and the error bar represents the standard error.

## Results

### Site-specifically labeled plasmid DNA is an optimal sample for smTIRF experiment

**Figure 1.**
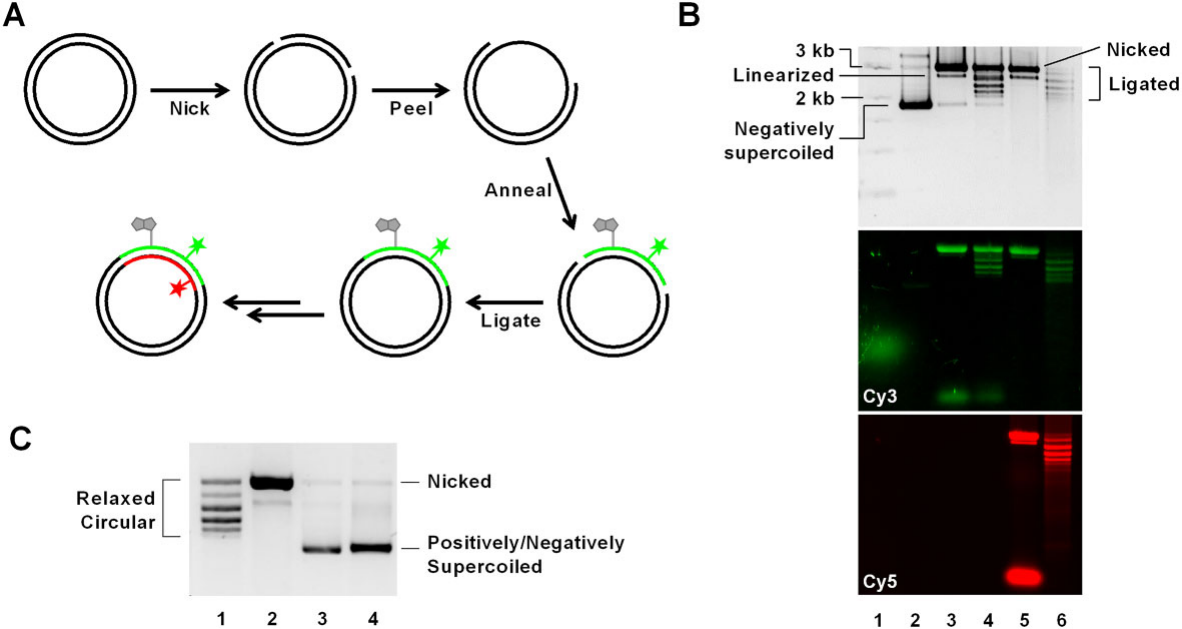
(**A**) Schematic representation of the plasmid preparation method. A plasmid (pUC19) is modified to carry two BbvCI recognition sites. The plasmid was prepared from *E. coli* after transformation. Two nicks are introduced into the plasmid using a nicking enzyme recognizing BbvCI sites. The small single-stranded DNA (ssDNA) fragment was peeled off at 95°C and was replaced by a labeled ssDNA of the same size. The nicks are ligated at the last step. The same process can be repeated on the other strand to introduce further modifications. (**B**) Gel electrophoresis images show plasmid samples at each preparation step. Lane 1, DNA ladder. Lane 2, native plasmid isolated from *E. coli*. The plasmid is negatively supercoiled and migrates faster than a linear DNA of equivalent molecular weight. Lane 3, plasmid nicked at two sites on one strand and annealed with an oligo with Cy3 and biotin modifications. The nicked plasmid migrates slower than the linear by-product. The second panel shows Cy3 signal, confirming the insertion of the Cy3-labeled oligo. Lane 4, ligated plasmid displaying discrete bands after ligation. Lane 5, plasmid nicked at two sites on the opposite strand of the product in lane 4 and annealed with a Cy5-labeled oligo. The bottom panel shows Cy5 signal, indicating the successful insertion of the Cy5-labeled oligo. Excess Cy5-labeled oligo appears as the bottom-most band. Lane 6, plasmid after final ligation, with nicked DNA removed by T5 exonuclease treatment. (**C**) A gel electrophoresis image showing Cy5-labeled plasmid samples of different supercoiling states. Lane 1, relaxed circular DNA. Lane 2, nicked DNA. Lane3, positively supercoiled DNA. Lane 4, negatively supercoiled DNA. Gel shows Cy5 signal.

Our aim was to achieve site-specific labeling of circular plasmid DNA with fluorophores and biotin so that we could introduce different supercoiling states while taking advantage of FRET analysis of surface-tethered DNA molecules for a detailed exploration of DNA–protein interactions and conformational changes. To site-specifically label circular plasmid DNA, we employed a nicking enzyme-based site-specific labeling strategy [31]. Initially, we cloned pUC19 plasmids containing two recognition sites for the nicking enzyme BbvCI, ∼50 bp apart from one another. After nicking the two sites, the small fragment that formed was evicted by elevating the temperature and replacing it with a single-stranded oligonucleotide labeled with a fluorophore and/or biotin while slowly cooling the mixture. Following ligation of the nicks, we successfully generated covalently closed circular DNA with labels (Fig. 1A). Through optimization at each step, we retrieved 40% of the initial plasmid DNA, sufficient for generating samples tailored for single-molecule experiments. We quantified the replacement efficiency, assessing how effectively the labeled single-stranded oligonucleotide replaced the native strand. By comparing the fluorescence intensity of the single-stranded oligonucleotide and circular DNA at equal concentrations, we determined that ∼60% of retrieved plasmid DNA had fluorescent labels (Supplementary Fig. S1). For further modifications, we repeated this process on the opposite strand. In the final step, we removed all the remaining single-stranded oligonucleotide and nonligated by-products by treating the mixture with T5 exonuclease (Fig. 1B). During the nicking and ligating steps, the supercoiling tension was released, generating relaxed circular DNA. Subsequently, we treated the plasmid DNA with gyrase or reverse gyrase to generate negatively or positively supercoiled DNA, respectively (Fig. 1C). To estimate the superhelical density (*σ*) of the resulting DNA, we performed two-dimensional gel electrophoresis (Supplementary Fig. S2). The plasmids treated with gyrase and reverse gyrase showed multiple topoisomers on the gel. A relaxed circular DNA sample was used as a control to estimate the linking number change (ΔLk), and the average superhelical densities were calculated. The negatively and positively supercoiled plasmids exhibited the average *σ* values of − 0.07 and + 0.03, respectively.

In subsequent experiments, we scrutinized the conditions for immobilization of plasmid DNA on a surface using TIRF microscopy. By incubating 200 pM of plasmid DNA for 5 min at room temperature, we successfully immobilized the DNA molecules with a suitable areal density (∼480 spots per imaging field of view) on a polymer-passivated surface using a biotin–NeutrAvidin interaction (Supplementary Fig. S3). Under these experimental conditions but without NeutrAvidin, the number of nonspecifically adsorbed spots was much smaller (∼55 per field of view). These observations demonstrate that our modified plasmid DNA is optimal for smTIRF measurements.

### CRISPR–Cas9: negative DNA supercoiling enhances R-loop formation in the presence of mismatch

**Figure 2.**
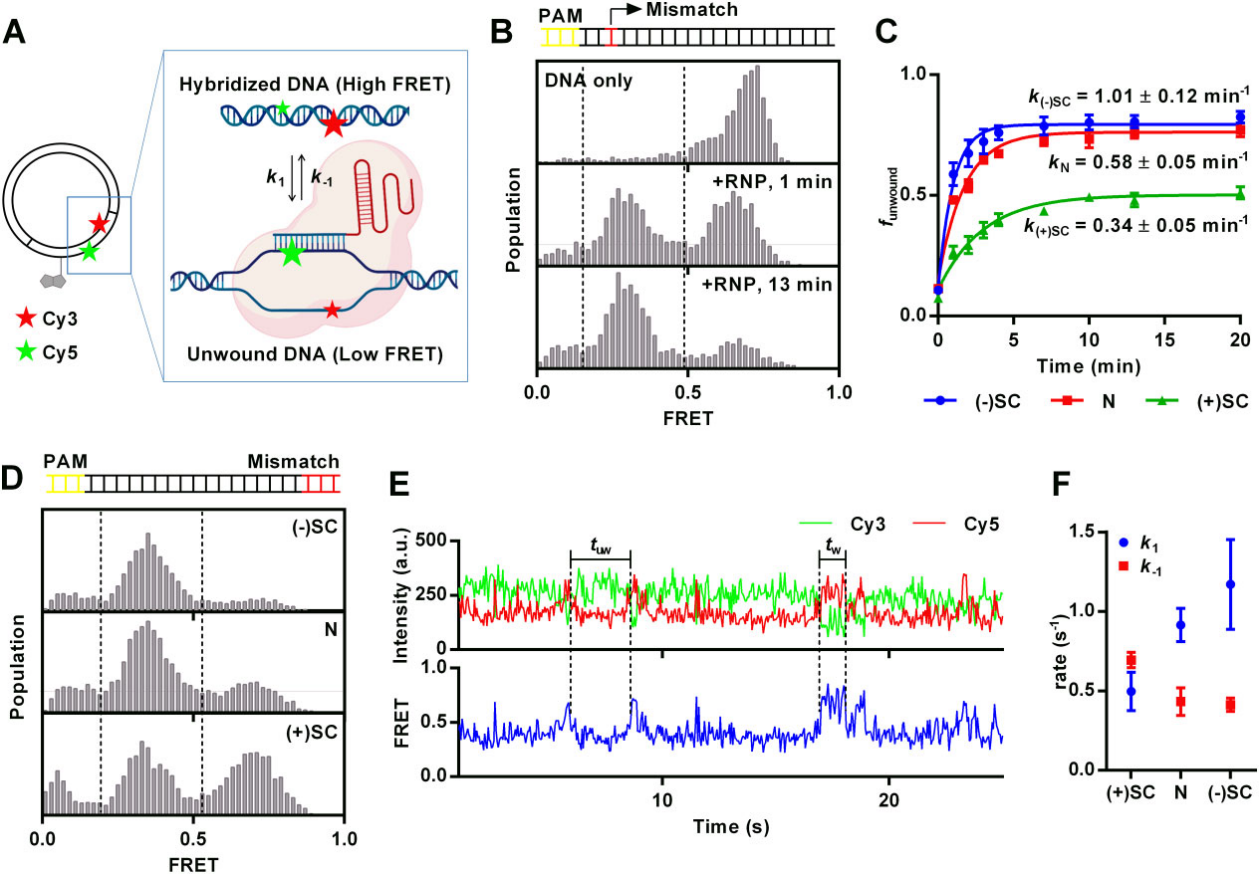
(**A**) Schematic representation of experimental design. Plasmid is labeled with a biotin 22 bp away from protospacer, with a donor (Cy3) and an acceptor (Cy5) positioned 10 bp apart on protospacer. The Cy3–Cy5 exhibits high FRET when DNA is fully hybridized, while low FRET indicates protospacer unwinding and R-loop formation by the dCas9–gRNA complex. (B, C) DNA unwinding when the gRNA has a single mismatch 3 bp from the PAM site. (**B**) FRET histograms of dCas9–gRNA binding to DNA constructs. Nicked plasmid was immobilized on a slide and 10 nM dCas9vgRNA was added. High FRET (∼0.6) was observed before the addition of dCas9–gRNA, and after 1 min of incubation, half of the DNA population shifted to low FRET (∼0.4). After 13 min, nearly the entire population exhibited low FRET. (**C**) Fraction of unwound DNA, *f*_unwound_, as a function of incubation time for negatively supercoiled [(−)SC], nicked (N), and positively supercoiled [(+)SC] plasmids. Approximately 5000 molecules were recorded over 20 s at each time point. Error bars represent standard error for *n* = 3. (D–F) DNA unwinding when the gRNA has three mismatches 18–20 bp away from the PAM site. (**D**) FRET histograms of dCas9–gRNA binding to negatively supercoiled [(−)SC], nicked (N), and positively supercoiled [(+)SC] plasmids. (**E**) Representative single-molecule intensity–time traces of Cy3 (green), Cy5 (red), and FRET efficiency (blue) showing DNA unwinding dynamics by dCas9–gRNA in nicked DNA. (**F**) Rate constants of the DNA unwinding and rewinding by dCas9–gRNA. The unwinding rate (*k*_1_) and rewinding rate (*k_−_*_1_) are indicated as blue circles and red squares, respectively. Over 500 data points are collected from over 100 molecules for each DNA sample. All triplicate data are provided in Supplementary Table S3. This figure contains elements created with BioRender.com.

In the first set of applications, we designed DNA constructs to investigate the impact of DNA supercoiling on DNA unwinding by CRISPR–Cas9. We previously developed an smFRET assay to probe DNA unwinding by dCas9–gRNA using short linear DNA molecules labeled with Cy3 (FRET donor) and Cy5 (FRET acceptor) [34, 36]. We implemented the same dual labeling system on circular plasmid DNA (Fig. 2A). The CRISPR–Cas9 target sequence was introduced into plasmid DNA. Subsequent modifications enabled the site-specific labeling of the target strand and the non-target strand with Cy3 and Cy5, respectively. In addition, biotin was incorporated 22 bp away from the protospacer to facilitate DNA immobilization. Before introducing dCas9–gRNA, the protospacer region of the DNA was fully base paired and exhibited high FRET efficiency (Fig. 2B). Following DNA incubation with dCas9–gRNA, the dCas9–gRNA unwound the protospacer region, displaying lower FRET efficiency. This shift in FRET efficiency reflects structural changes in DNA induced by the interaction of dCas9–gRNA with the protospacer region, providing a dynamic readout of CRISPR–Cas9 unwinding activity on the plasmid DNA construct.

To further investigate the effects of DNA supercoiling on DNA unwinding activity of dCas9–gRNA, we induced negative and positive supercoiling in the labeled plasmid DNA using gyrase and reverse gyrase, respectively. We also employed nicked DNA as a “zero-supercoiling-level” control, achieved through a specific nicking enzyme treatment. The nick site was positioned ∼1000 bp away from the protospacer to ensure that its influence on CRISPR–Cas9 was limited to the effects of supercoiling relief rather than directly affecting interactions with the protein. Single-molecule FRET histograms in the absence of dCas9–gRNA showed highly similar distributions between the three plasmids with distinct topological states (Supplementary Fig. S4). We initially incubated the plasmids with dCas9–gRNA, which perfectly matched the DNA target sequence. DNA unwinding was so rapid in both negatively supercoiled and nicked DNA that it was difficult to discern any difference in rate (Supplementary Fig. S5).

Next, to examine the effects of DNA supercoiling on off-target binding and unwinding by CRISPR–Cas9, we introduced a single nucleotide mismatch between the target sequence and the gRNA in the PAM-proximal region. Using this dCas9–gRNA complex, the negatively supercoiled DNA displayed the most pronounced shift in FRET populations from high to low, compared to the nicked DNA and positively supercoiled DNA under identical conditions (Fig. 2C). To quantify the unwinding activity across three different DNA constructs, we calculated the fraction of the unwound state, *f*_unwound_, by dividing the population of low FRET by the sum of low FRET and high FRET populations and plotted *f*_unwound_ over time after the addition of 10 nM of dCas9–gRNA. The data were fitted to a one-phase association model using equation (4) to obtain the rate constant (*k*).


(4) \begin{eqnarray*}
{{y}} = {y_0} + \left( {{\rm plateau} - {y_0}} \right)\left( {1 - {{\rm e}^{ - kx}}} \right).
\end{eqnarray*}

The negatively supercoiled DNA exhibited approximately a two- and three-fold higher rate constant (*k*) compared to nicked DNA and positively supercoiled DNA, respectively. These results suggest that negative supercoiling enhances unwinding of the target DNA by dCas9–gRNA containing a PAM-proximal mismatch. Additionally, the negatively supercoiled and nicked DNA substrates reached a plateau at 0.79 and 0.76, respectively, indicating higher levels of product accumulation than those of positively supercoiled DNA, which plateaued at 0.50. Together, these observations suggest that positive supercoiling kinetically hinders the unwinding process, likely due to the increased torsional strain on the DNA helix impacting the efficiency of DNA strand separation (Fig. 2C) [37, 38].

Next, we modified the gRNA to introduce three mismatched base pairs between the DNA target sequence and gRNA, spanning positions 18–20, counting from the PAM site. Upon addition of the dCas9–gRNA, we measured FRET efficiency at equilibrium (Fig. 2D). Negatively supercoiled DNA showed the highest population ratio of DNA unwound (*f*_unwound_ = 0.77), followed by nicked DNA (*f*_unwound_ = 0.67) and then positively supercoiled DNA (*f*_unwound_ = 0.45) (Supplementary Table S3). Additionally, we observed dynamic FRET fluctuations, suggesting ongoing dynamic DNA conformational changes (Fig. 2E). To further analyze the dynamics, we applied hidden Markov models using ebFRET [35], which identified two distinct conformational trajectories characterized by high and low FRET states. We quantified the dwell times for each of these FRET states. The resulting data were transformed into the complement of the cumulative distribution function (1 − CDF) and fitted to a first-order exponential decay model, allowing us to calculate the DNA unwinding and rewinding rate constants, *k*_1_ and *k*_−1_. For nicked DNA, the *k*_1_ and *k*_−1_ were ∼0.9 and 0.4 s^−1^, respectively (Fig. 2F and Supplementary Table S3). These values deviate in the expected direction from the bulk solution kinetic data that gave ∼1.6 s^−1^ for unwinding and ∼0.1 s^−1^ for rewinding when the gRNA perfectly matches the target DNA sequence [39]. The unwinding rate constant *k*_1_ appeared higher in the order negatively supercoiled DNA > nicked DNA > positively supercoiled DNA. In contrast, the rewinding rate constant *k*_−1_ appeared similar for nicked DNA and negatively supercoiled DNA but was highest in positively supercoiled DNA (Fig. 2F). These results indicate that negatively supercoiled DNA exhibits a higher degree of intrinsic unwinding when mismatches are present in the dCas9–gRNA due to faster unwinding and slower rewinding. This demonstrates that negative supercoiling facilitates DNA unwinding by dCas9–gRNA in the presence of mismatches [37, 40, 41].

### MutS: DNA supercoiling stabilizes the interaction between MutS and a mismatched base pair

**Figure 3.**
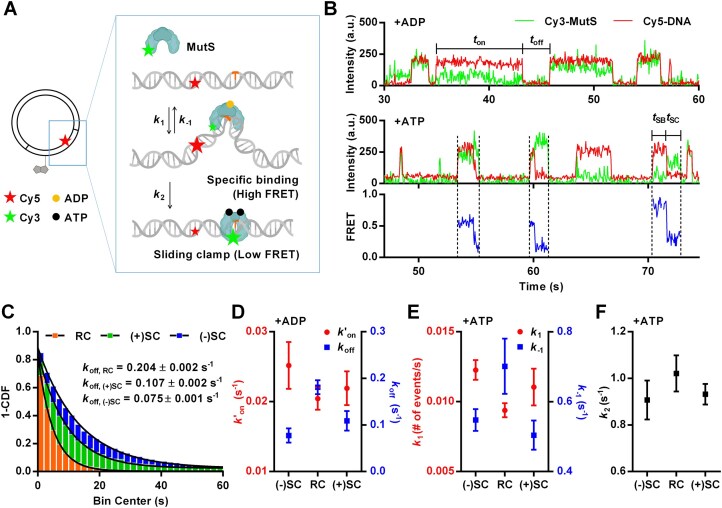
(**A**) Schematic representation of experimental design. Plasmid is labeled with a biotin and a Cy5, 26 and 8 bp away from a single GT mismatch (colored as orange), respectively. Cy3-labeled, ADP-bound MutS binding to a mismatch causes high FRET between Cy3 on MutS and Cy5 on DNA. MutS undergoes an ADP–ATP exchange and forms a sliding clamp, characterized by low FRET. (**B**) Representative single-molecule time traces of Cy3 signal (green), Cy5 signal (red), and FRET efficiency (blue), showing MutS binding to a relaxed circular DNA containing a GT mismatch supplemented with 1 mM of ADP (upper panel) and 1 mM of ATP (lower panel). FRET efficiency shows heterogeneities between individual MutS binding events due to the presence of both mono- and di-labeled MutS. (**C**) MutS dissociation rate (*k*_off_) in the presence of ADP. Binding time (*t*_on_) is collected and transformed into 1 − CDF graph. This graph is fitted into a single exponential decay function to determine the *k*_off_. Relaxed circular DNA (RC) showed a dissociation rate of 0.204 ± 0.002 s^−1^, positively supercoiled plasmid [(+)SC] showed 0.107 ± 0.002 s^−1^, and negatively supercoiled plasmid [(−)SC] exhibited 0.075 ± 0.001 s^−1^. Approximately 500 events were collected from 200 molecules for each DNA sample. All triplicate data are provided in Supplementary Table S4. (**D**) Pseudo-first-order association rate (*k′*_on_) and dissociation rate (*k*_off_) of MutS binding to a GT mismatch in the presence of ADP. The error bars represent standard error for *n* = 3. (**E**, **F**) The rate constants, *k*_1_, *k*_−1_, and *k*_2_ in the presence of ATP. The error bars represent standard error for *n* = 4 for negatively supercoiled DNA and relaxed circular DNA, and *n* = 3 for positively supercoiled DNA. Each data point was collected from ∼400 molecules. This figure contains elements created with BioRender.com.

For an additional demonstration of the versatility of our approach, we extended our study to include *E. coli* MutS, a crucial protein in the DNA MMR process [42–44]. MutS recognizes and binds to a mismatched base pair while bound to ADP [45]. Once bound to a mismatched base pair, MutS exchanges ADP for ATP and undergoes a conformational change, resulting in the formation of a sliding clamp [46]. The sliding clamp formation is essential for initiating the MMR pathway, as it recruits other repair factors [47–49]. Our study focuses on two key aspects of MutS: its binding affinity for the mismatch region and its transformation into the sliding clamp configuration. Here, we examined MutS behavior with plasmid DNA containing a GT mismatch. This specific mismatch was chosen due to its known propensity to exhibit significant MutS binding and sliding clamp formation [50–52]. To facilitate this study, the plasmid DNA was labeled with Cy5, positioned 8 bp away from the mismatch site (Fig. 3A). This labeling position was designed to characterize the interactions between Cy3-labeled MutS and DNA via FRET [51, 53]. This approach allowed us to observe high FRET efficiency when MutS bound specifically to the mismatched base pair (Fig. 3B). Moreover, these MutS binding events were often followed by sliding clamp formation in the presence of ATP, characterized by a transition to low FRET [54]. Finally, to investigate the effect of DNA supercoiling on MutS activity, we introduced negative and positive supercoiling into the Cy5-labeled, GT mismatch-containing plasmid DNA using the same approach as in our CRISPR–Cas9 experiments. To avoid potential interference from nicks affecting MutS [55, 56], we used relaxed circular DNA as the control instead of nicked DNA.

To determine the binding affinity, we incubated the plasmid DNA with MutS and ADP, which allowed us to exclusively observe high-affinity MutS binding without sliding clamp formation [54]. As expected, we observed a repetitive pattern of MutS binding to and dissociating from the mismatched region. To quantitatively analyze these interactions, we measured both the dwell time (*t*_on_), representing the duration of MutS binding to the mismatched region as observed through the high FRET value, and the waiting time (*t*_off_), indicating the interval between successive binding events. The collected data were transformed into a 1 − CDF and fitted to a first-order exponential decay function (Fig. 3C). From this analysis, we derived kinetic parameters, including the pseudo-first-order association rate (*k′*_on_) and dissociation rate (*k*_off_). We observed that negatively supercoiled DNA exhibited a *k*_off_ value ∼2.7 times lower than that of relaxed circular DNA. Positively supercoiled DNA also showed slower dissociation, by a factor of 1.4, compared to relaxed circular DNA. The trend for *k′*_on_ between different DNA samples was the opposite, though the difference was more subtle. Negatively supercoiled DNA showed 1.2 times faster MutS binding compared to relaxed circular, while positively supercoiled DNA had a similar association rate to that of relaxed circular DNA (Fig. 3D and Supplementary Table S4). Overall, our data suggest that both positive and negative supercoiling of DNA enhance the stability of the MutS–mismatch interaction in the presence of ADP once binding occurs. However, the initial mismatch binding occurs with similar kinetics between supercoiled DNA and relaxed circular DNA.

Following our observations under ADP conditions, we next examined the impact of ATP on MutS–mismatch interaction dynamics. Under ATP conditions, we introduced three rate constants to account not only for MutS binding affinity but also for its subsequent ATP binding-induced conformational change to form a sliding clamp (Fig. 3A). We defined the rate of MutS binding to the mismatch region as *k*_1_, the dissociation rate as *k*_−1_, and the conformational change to the sliding clamp from the initial mismatch-bound state as *k*_2_. Once MutS forms a sliding clamp, subsequent protein dynamics become difficult to analyze because the low FRET state encompasses multiple scenarios, representing both the sliding clamp conformation and the potential movement of MutS away from the mismatch region. The termination of the low FRET state, coupled with the absence of the green signal, signifies either the dissociation of MutS from the DNA or its sliding away from the mismatch region beyond the evanescent field of total internal reflection excitation. Therefore, we focused our analysis on sliding clamp formation rather than beyond.

To estimate *k*_1_, we counted the number of MutS–mismatch binding events and calculated the number of binding events per second. To determine *k*_2_ and *k*_−1_, we measured two quantities, MutS dwell time on the mismatch (as a specific binding mode, represented by the high FRET state) and the branching ratio between sliding clamp formation and dissociation (see the “Materials and methods” section). This comprehensive approach allowed us to dissect the dynamic aspects of MutS–mismatch interactions in the presence of ATP. We found that negatively supercoiled DNA exhibited a *k*_1_ value ∼1.3 times higher than that of relaxed circular DNA in the presence of ATP (Fig. 3E), similar to what we observed in the presence of ADP. We obtained very similar *k*_1_ values for positively supercoiled DNA and relaxed circular DNA. However, negatively and positively supercoiled DNA showed a dissociation rate (*k*_−1_) ∼1.3 times lower than that of relaxed circular DNA. The rate of sliding clamp formation, *k*_2_, did not change significantly with either negative or positive supercoiling (Fig. 3F). Overall, our data suggest that the higher apparent mismatch affinity for supercoiled DNA compared to relaxed DNA is a conserved property even when ATP-induced sliding clamp formation is permitted, and that sliding clamp formation kinetics do not depend on the supercoiling state.

## Discussion

Utilizing our plasmid internal-DNA modification method, we successfully generated supercoiled plasmids dually labeled with a fluorophore and biotin with or without a mismatch at specific locations. This advance allowed us to employ smTIRF microscopy to investigate the impact of DNA supercoiling on DNA–protein interactions across dozens to hundreds of single molecules in parallel. Our experiments with CRISPR–Cas9 revealed a significant preference for negative supercoiling by the dCas9–gRNA complex during DNA unwinding. This observation aligns with previous studies, such as those using rotor bead tracking [37] and optical tweezer methods [40, 57]. For instance, the rotor bead tracking assay demonstrated that negative torque on DNA can induce both half- and fully unwound states of the DNA, which are required for Cas9 cleavage [39, 58], suggesting a role for supercoiling in facilitating CRISPR–Cas9 activity. Similarly, studies using optical tweezers found that more nonspecific binding occurs in negatively supercoiled or strongly stretched DNA [40, 57]. However, unlike these previous methods, which investigated DNA binding and unwinding by Cas9–gRNA while altering the DNA supercoiling status, our FRET-based approach measured the real-time kinetics of DNA unwinding at a fixed supercoiling state, providing a more direct analysis of the unwinding process. Furthermore, while previous optical tweezer-based studies required very high levels of negative supercoiling (e.g. *σ* = −0.15 to −0.7) to observe effects on CRISPR–Cas9 activity [40]—well beyond the physiological range (*σ* ≈ −0.07)—our system demonstrated that supercoiling influences DNA unwinding kinetics even within a physiologically relevant range (*σ* = −0.07 and *σ* = 0.03) [6, 8, 9, 11, 59–62]. This underscores the biological relevance of our findings and suggests that DNA topology *in vivo* could play a more nuanced and significant role in regulating CRISPR–Cas9 specificity than previously recognized.

In our study of MutS, we made a novel observation suggesting that DNA supercoiling could influence MutS binding affinity for the mismatch region. Through dwell time analysis, we discovered that supercoiled DNA stabilized MutS binding to the mismatch region in the presence of ADP. One hypothesis is that DNA supercoiling aids DNA conformational dynamics, including DNA bending at the mismatch, which may be captured or induced by MutS binding. The mismatched region, having different deformation energy compared to the homoduplex, allows MutS to distinguish the mismatch region by readily bending and kinking it [45, 63–66]. DNA supercoiling further enhances these structural changes, such as kinking or bending [59, 67–71], and the increased ability to deform DNA structure provides a rationale for why supercoiled DNA exhibits a longer binding time with MutS [45, 63–66, 72]. Since the MutS ATP-induced conformational change into a sliding clamp is coupled with DNA unbending, it was formally possible that DNA supercoiling could affect the efficiency of MutS sliding clamp formation. However, we observed only a subtle difference in the rate of MutS sliding clamp formation between different supercoiling states. These results suggest that DNA supercoiling may play a role in the MutS’s initial recognition step and/or maintain the bent state when MutS is bound to DNA but is unlikely to influence the step that results in sliding clamp formation. The conformational changes of MutS include multiple steps such as DNA unbending and ADP–ATP exchange [46, 73]. These multiple steps potentially obscure any effects of DNA supercoiling on DNA unbending by MutS.

Previously, we found that MMR efficiency *in vivo* is highly sequence dependent, influenced not only by the mismatch itself but also by the adjacent sequence context [52]. We also found that MutS binding affinity for various mismatch sequence configurations *in vitro* correlates with repair efficiency *in vivo* and these differences originate from the altered DNA dynamics of the mismatch-containing sequences [52, 72]. Our current findings, showing that MutS binds to the same mismatch differently depending on the supercoiling state, support our previous conclusion that MutS senses the altered properties of the DNA that a mismatched nucleotide induces within the duplex structure, which may explain how it identifies mismatches in different sequence contexts [52, 72]. Further investigations are warranted to unravel the intricate details of how MutS senses these differences.

While our findings provide valuable insights into the effects of DNA supercoiling on CRISPR–Cas9 and MutS interactions, we can enhance our current platform to better study supercoiling dynamics as they occur within living cells. In cells, DNA exhibits local supercoiling that dynamically changes in response to DNA metabolic processes such as transcription and replication [6, 8, 9, 11, 59–62]. In eukaryotes, DNA is wrapped around histone octamers, producing a slightly negatively supercoiled chromatin structure [74]. The relatively unconstrained DNA within chromatin can acquire negative supercoiling up to an upper limit of *σ* = −0.07 due to transcription *in vivo* [75]. The negative supercoiling generated in our plasmid system, estimated to be of density *σ* of −0.07 according to our 2D gel electrophoresis, closely mirrors the supercoiling states typically observed in cells. Since the supercoiling effect caused by transcription varies depending on factors such as the distance from the promoter and the strength of the promoter itself [8], future studies could introduce a promoter of interest into the plasmid to better mimic cellular conditions and further explore these effects. Given that transcription-induced supercoiling can diffuse across a distance of over 1.5 kb [8], our plasmid (2.8 kb) is well suited to accommodate and study these effects.

In conclusion, our platform for studying DNA supercoiling effects in DNA–protein interactions offers a promising approach for investigating various DNA-processing enzymes that alter DNA conformations [76, 77] or are influenced by DNA flexibility, such as transcription factors [78]. Additionally, our labeling strategy enables the generation of other substrates, including mismatch repeat assemblies, which could be applied to optical tweezers or other force spectroscopy techniques. This versatility expands the platform’s application beyond DNA repair, making it a valuable tool for exploring the dynamic interactions between DNA and diverse enzymes. Indeed, we recently used this platform to examine the effects of DNA supercoiling on the interplay between R-loop formation and transcription [79].

## Supplementary Material

gkaf581_Supplemental_File

## Data Availability

The MATLAB scripts underlying this article are available in Zenodo Digital Repository, at https://doi.org/10.5281/zenodo.13987136. The gene maps of plasmid are available in Zenodo Digital Repository, at https://doi.org/10.5281/zenodo.13988016. The other data underlying this article will be shared on request to the corresponding author.
