# Correction to ‘A high-throughput single-molecule platform to study DNA supercoiling effect on protein–DNA interactions’

**DOI:** 10.1093/nar/gkaf850

**Published:** 2025-08-19

This is a correction to: Huijin Lee, Fahad Rashid, Jihee Hwang, James A London, Richard Fishel, James M Berger, Sua Myong, Taekjip Ha, A high-throughput single-molecule platform to study DNA supercoiling effect on protein–DNA interactions, *Nucleic Acids Research*, Volume 53, Issue 12, 8 July 2025, gkaf581, https://doi.org/10.1093/nar/gkaf581

The authors initially omitted two important references, which have now been added as citations [80] and [81]. These articles are discussed and cited in the Introduction and Materials and Methods sections.

This change does not affect the results, discussion and conclusions presented in the article. The published article has been updated.

## References

[80] Gu M, Berrido A, Gonzalez WG et al. Fluorescently labeled circular DNA molecules for DNA topology and topoisomerases. Sci Rep. 2016; 6:36006 10.1038/srep36006. 27796331 PMC5087112

[81] Wang Y, Rakela S, Chambers JW et al. Kinetic Study of DNA Topoisomerases by Supercoiling-Dependent Fluorescence Quenching. ACS Omega. 2019; 4:18413–22. 10.1021/acsomega.9b02676. 31720544 PMC6844113

