# Supplementary material for: A high-throughput single-molecule platform to study DNA supercoiling effect on protein–DNA interactions
Source: Nucleic Acids Res. 2025 Jun 28;53(12):gkaf581. doi: 10.1093/nar/gkaf581 (PMC12205362; doi:10.1093/nar/gkaf581)
Supplement: gkaf581_Supplemental_File [file gkaf581_supplemental_file.pdf]

SUPPLEMENTARY DATA

A high throughput single molecule platform to study DNA supercoiling effect on protein-DNA interactions

Huijin Lee[1, 2], Fahad Rashid[2], Jihee Hwang[3,4], James A. London[5], Richard Fishel[5], James M. Berger[2], Sua Myong[3, 4] and Taekjip Ha[1, 2, 4, *]

[1]Howard Hughes Medical Institute and Programs in Cellular and Molecular Medicine, Boston Children's Hospital, Boston, Massachusetts, 02115, USA

[2]Department of Biophysics and Biophysical Chemistry, Johns Hopkins School of Medicine, Baltimore, Maryland, 21205, USA

[3]Programs in Cellular and Molecular Medicine, Boston Children's Hospital, Boston, Massachusetts, 02115, USA

[4]Department of Pediatrics, Harvard Medical School, Boston, Massachusetts, 02115, USA

[5]Department of Cancer Biology and Genetics, The Ohio State University Wexner Medical Center, Columbus, Ohio, 43210, USA

* To whom correspondence should be addressed. Tel: +1 217 398 0865; Email: Taekjip.Ha@childrens.harvard.edu

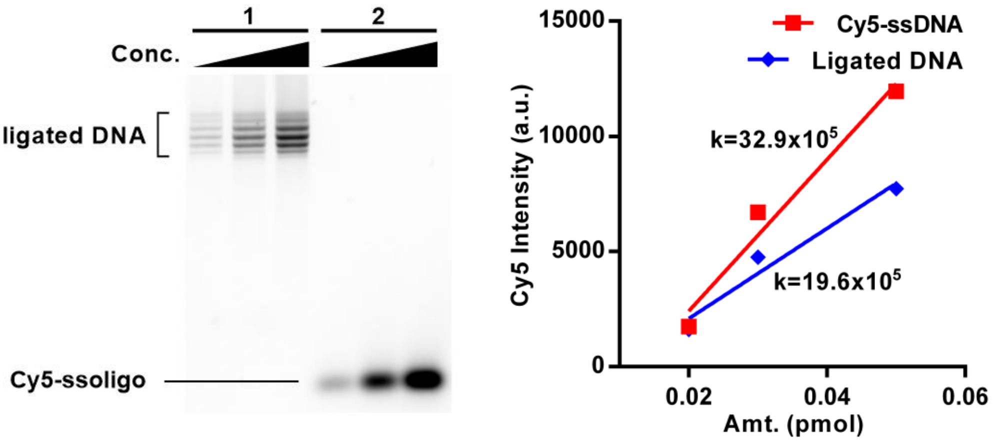

Supplementary Figure S1: Evaluation of Cy5-labeled Oligo Incorporation into Plasmid DNA. A portion of the plasmid DNA was replaced with Cy5-labeled single-stranded oligo DNA using the strand replacement method described in main text. The plasmids and Cy5-labeled oligo were loaded onto a 1% agarose gel at three different concentrations (Left). To determine how efficiently the Cy5-labeled oligo was incorporated into the plasmid, the fluorescence intensity of ligated plasmid DNA was compared with that of the input Cy5-labeled oligo at each concentration. A standard curve was generated by plotting Cy5 fluorescence intensity against DNA amount (Right). The slope (k) of each curve reflects the fluorescence signal per unit DNA, allowing for relative efficiency comparisons. The ligated DNA showed a lower slope ($k = 19.6 \times 10^5$) than the input Cy5-ssDNA ($k = 32.9 \times 10^5$), corresponding to an estimated incorporation efficiency of approximately 60%. This suggests that a substantial fraction of the input Cy5-labeled oligo was successfully incorporated into the plasmid, while the remainder likely remained unligated or lost during purification.

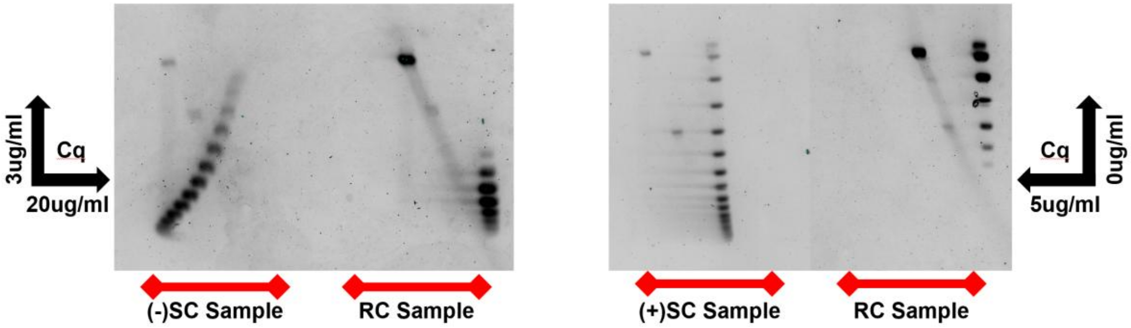

Supplementary Figure S2: Quantification of the DNA supercoiling level by two-dimensional (2D) gel electrophoresis. The negatively supercoiled DNA ((-)SC) and relaxed circular DNA (RC) on the left. The linking number change (ΔLk) of the negatively supercoiled plasmid ranges from -11 to -23, with a median ΔLk of -18. Based on this value and the length of the plasmid, the calculated superhelical density (σ) is approximately -0.07. The positively supercoiled DNA ((+)SC) and relaxed circular DNA (RC) on the right. The ΔLk of the positively supercoiled plasmid ranges from -2 to +11, with a median ΔLk of +7. Based on this value and the length of the plasmid, the calculated superhelical density (σ) is approximately +0.03. Cq: Chloroquine.

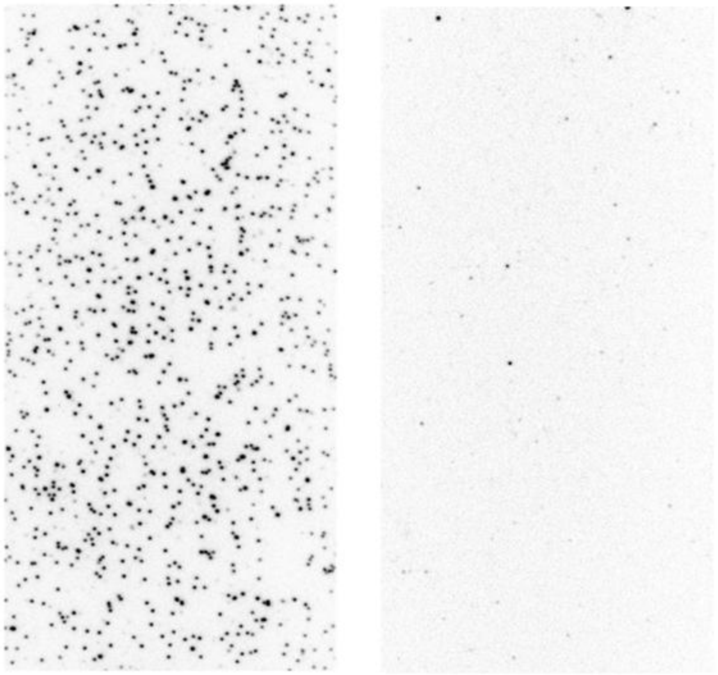

Supplementary Figure S3: Specific binding and non-specific binding of site-specifically labeled plasmids. Cy5 labeled DNA (200 pM) was incubated on the NeutrAvidin treated (left) verse non-treated (right) surfaces for 10 min at room temperature and imaged using Cy5 excitation/emission.

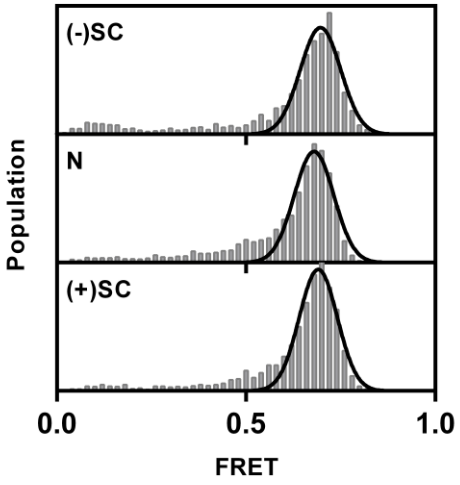

Supplementary Figure S4: FRET histograms of each plasmid. Each histogram was fitted into Gaussian distribution. The mean value was 0.70 for negatively supercoiled DNA, 0.68 for nicked DNA and 0.69 for positively supercoiled DNA.

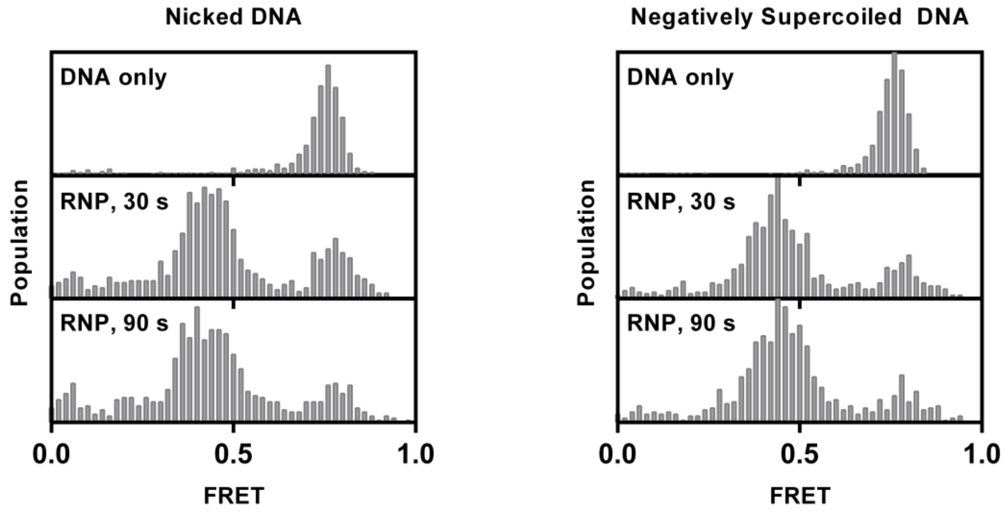

Supplementary Figure S5: FRET histograms of nicked DNA and negatively supercoiled DNA upon addition of dCas9-gRNA, which perfectly matches the target DNA sequence. Approximately 10 fields of view were imaged immediately after dCas9-gRNA addition (30 seconds), and again after 1 minute. $f_{unwound}$ values were 0.02, 0.56, and 0.55 for nicked DNA, and 0.06, 0.57, and 0.60 for negatively supercoiled DNA in the DNA-only, 30 seconds incubation, and 90 seconds incubation conditions, respectively. Histograms were generated from more than 500 molecules.

Supplementary Table S1. Single-stranded oligo nucleotides sequence

		DNA sequence
Rloop	Top	5′-Phos-TCAGCCCAGCGTCTCATCTTTATACATCAGCAGAGATTTCTGCTGTGCAACC
	Bottom	5′-Phos-TGAGG**T**TGCACAGCAGAAATCTCTGCTGATGTATAAAGATGAGACGCTGGGC
MutS	Top	5′-Phos-TCAGCT**T**AATACGACTCACTATAGGCCAATACA**G**GAGCTTCATCC
	Bottom	5′-Phos-TGAGGATGAAGCTCTTGTATTGGCCTATAGTGAGTCGTATTA AGC

* T: amino-dT labeled with Cy5, T: amino-dT labeled with Cy3, G: GT mismatch, **T**: Biotinylated T

Supplementary Table S2. Crispr RNA sequence

Mismatch position from PAM	RNA sequence
3	rGrArUrGrUrArUrArArArGrArUrGrArGrArArGrCrGrUrUrUrUrArGrArGrCrUrArUrGrCrU
18-20	rArGrCrGrUrArUrArArArGrArUrGrArGrArCrGrCrGrUrUrUrUrArGrArGrCrUrArUrGrCrU

Supplementary Table S3. Rate constants of DNA unwinding and rewinding by dCas9-RNP. They are expressed in s^{-1}.

	k_1				
	**Exp 1**	**Exp 2**	**Exp 3**	**mean**	**standard error**
**(-)SC**	1.070	0.952	1.488	1.170	0.163
**N**	0.796	0.953	0.995	0.915	0.060
**(+)SC**	0.629	0.391	0.471	0.497	0.070
	k_{-1}				
	**Exp 1**	**Exp 2**	**Exp 3**	**mean**	**standard error**
**(-)SC**	0.394	0.384	0.461	0.413	0.024
**N**	0.398	0.532	0.368	0.432	0.050
**(+)SC**	0.664	0.669	0.750	0.694	0.028
	$F_{unwound}$				
	**Exp 1**	**Exp 2**	**Exp 3**	**mean**	**standard error**
**(-)SC**	0.736	0.798	0.790	0.775	0.019
**N**	0.652	0.662	0.693	0.669	0.012
**(+)SC**	0.456	0.424	0.478	0.453	0.016

Supplementary Table S4. Rate constants of MutS association and dissociation in the presence of ADP.

	k_{off}				
	**Exp 1**	**Exp 2**	**Exp 3**	**mean**	**standard error**
**(-)SC**	0.0749	0.0521	0.1049	0.0773	0.0153
**(+)SC**	0.1066	0.0738	0.1462	0.1089	0.0209
**RC**	0.2043	0.1532	0.1852	0.1809	0.0149
	k'_{on}				
	**Exp 1**	**Exp 2**	**Exp 3**	**mean**	**standard error**
**(-)SC**	0.0244	0.0198	0.0313	0.0252	0.0033
**(+)SC**	0.0223	0.0176	0.0259	0.0219	0.0024
**RC**	0.0208	0.0175	0.0230	0.0204	0.0016
	k_{on}				
	**Exp 1**	**Exp 2**	**Exp 3**	**mean**	**standard error**
**(-)SC**	1.220E+07	9.909E+06	1.566E+07	1.259E+07	1.673E+06
**(+)SC**	1.115E+07	8.776E+06	1.295E+07	1.096E+07	1.209E+06
**RC**	1.041E+07	8.752E+06	1.148E+07	1.021E+07	7.948E+05
